# Changes in the Distribution of the ****α****3 Na^+^/K^+^ ATPase Subunit in Heterozygous Lurcher Purkinje Cells as a Genetic Model of Chronic Depolarization during Development

**DOI:** 10.1155/2014/152645

**Published:** 2014-02-27

**Authors:** Rebecca McFarland, Hadi S. Zanjani, Jean Mariani, Michael W. Vogel

**Affiliations:** ^1^Maryland Psychiatric Research Center, Department of Psychiatry, University of Maryland School of Medicine, P.O. Box 21247, Baltimore, MD 21228, USA; ^2^Department of Biology, University of Maryland Baltimore County, Baltimore, MD 21201, USA; ^3^Université Pierre et Marie Curie-P6, UMR7102, 75005 Paris, France; ^4^Institut de la Longévité, Hôpital Charles Foix, 94205 Ivry-Sur-Seine, France

## Abstract

A common assumption of excitotoxic mechanisms in the nervous system is that the ionic imbalance resulting from overstimulation of glutamate receptors and increased Na^+^ and Ca^++^ influx overwhelms cellular energy metabolic systems leading to cell death. The goal of this study was to examine how a chronic Na^+^ channel leak current in developing Purkinje cells in the heterozygous Lurcher mutant (+/*Lc*) affects the expression and distribution of the **α**3 subunit of the Na^+^/K^+^ ATPase pump, a key component of the homeostasis system that maintains ionic equilibrium in neurons. The expression pattern of the catalytic **α**3 Na^+^/K^+^ ATPase subunit was analyzed by immunohistochemistry, histochemistry, and Western Blots in wild type (WT) and +/*Lc* cerebella at postnatal days P10, P15, and P25 to determine if there are changes in the distribution of active Na^+^/K^+^ ATPase subunits in degenerating Purkinje cells. The results suggest that the expression of the catalytic **α**3 subunit is altered in chronically depolarized +/*Lc* Purkinje cells, although the density of active Na^+^/K^+^ ATPase pumps is not significantly altered compared with WT in the cerebellar cortex at P15, and then declines from P15 to P25 in the +/*Lc* cerebellum as the +/*Lc* Purkinje cells degenerate.

## 1. Introduction

The Na^+^/K^+^ ATPase pump (Na/K pump) in neurons plays a key role in maintaining the transmembrane electrical gradient that is critical for normal function. The mature pump resides in the plasma membrane and exports 3 Na^+^ ions for every two K^+^ ions it imports at a cost of 1 ATP molecule, resulting in a net outward, hyperpolarizing current [1]. In healthy cerebellar Purkinje cells, increases in the expression levels of the Na/K pump during postnatal development are associated with the gradual hyperpolarization of the Purkinje cell membrane potential [2, 3]. All Na/K pumps contain an alpha (*α*) and a beta (*β*) unit, though in some cell types the pump contains an additional FXYD protein [4, 5]. There are at least 4 isoforms of the alpha subunit and 3 isoforms of the beta subunit. The *α* subunit is the catalytic unit and is responsible for binding of Na^+^ and K^+^ ions, ATP, and ouabain (an inhibitor). The *β* subunit plays a crucial role in the structure and maturation of the Na/K pump, including, for example, aiding in the tracking of the *α* subunit from the ER to the plasma membrane [6]. Cerebellar Purkinje cells exclusively express the *α*3 and *β*1 subunits, but some other cerebellar neurons (e.g., basket cells) or structures (e.g., granule cell layer glomeruli) also express the *α*3 subunit [7]. The FXYD family protein, FXYD1 (Phospholemman), is also expressed in Purkinje cells and the molecular layer of the cerebellum [8], but this protein was not analyzed in this study.

Na/K ATPase activity in neurons may contribute as much as 240 pA to the resting membrane potential and pump activity is increased as a function of neuronal activity [3]. Changes in Na/K pump activity are also associated with the adaptive responses of neurons to hypoxia, ischemia, and cell death [5, 9]. However, whether pump activity is increased or decreased in response to acute models of cellular injury appears to depend on the cell type or injury model [10–13]. There is evidence that Na/K pump activity is directly regulated by a variety of cellular second messenger signaling systems. For example, nitric oxide (NO)/cGMP intracellular signaling can downregulate Na/K pump activity [10]. Conversely, in cerebellar Purkinje cells, carbon monoxide (CO) and glutamate can act through cGMP to promote persistent increases in Na/K pump activity [14]. Under excitotoxic conditions, peroxynitrite and reactive oxygen can react with the Na/K pump to deactivate it [15, 16]. In general, excitotoxic mechanisms in neurons are associated with overstimulation of glutamate receptors resulting in an increased Na^+^ and Ca^++^ influx that overwhelms cellular energy metabolic systems leading to cell death [17, 18]. Both increases and decreases in Na/K pump activity may be theoretically associated with neuroprotective or cell-death promoting activities. In chronically depolarized neurons, increased Na/K pump activity could be seen to reduce intracellular Na^+^ levels to help restore cellular homeostasis, but the increased demand for ATP could be deleterious. Alternatively, decreased Na/K pump activity in injured cells may allow for increased intracellular Na^+^, but the decreased demand for ATP from less active Na/K pumps may be neuroprotective.

A recent study of HEK293 cells expressing *δ*2 glutamate receptors (GluR*δ*2) with the Lurcher mutation, GluR*δ*2^*Lc*^, found that ectopic expression of the mutant receptor in cultured cells causes a decline in ATP levels [19]. The *Lc* mutation in GluR*δ*2 turns the receptor into a constitutively open membrane channel that chronically depolarizes the cells expressing the mutant receptor [20]. In the HEK293 *in vitro* studies, the authors speculate that over activation of the Na/K pump in response to the chronic Na^+^ leak may consume cellular ATP levels leading to cell death [19, 21]. The purpose of this study is to examine how a chronic Na^+^ leak current mediated by the mutant GluR*δ*2^*Lc*^ receptor in developing Purkinje cells in the heterozygous Lurcher (+/*Lc*) mouse mutant affects the cellular distribution of *α*3 subunits and active Na/K pumps.

The gene for the GluR*δ*2 receptor was first identified based on its homology to NMDA and AMPA glutamate receptors, but the receptor does not bind to most glutamate agonists or antagonists, nor does it appear to carry a membrane current under normal circumstances. GluR*δ*2 is preferentially expressed in cerebellar Purkinje cells, and in the +/*Lc* mutant, cerebellar Purkinje cells become chronically depolarized at the end of the first postnatal week when GluR*δ*2 receptors are inserted at Purkinje cell-parallel fiber synapses. The majority of +/*Lc* Purkinje cells degenerate during the first month of postnatal life starting at the end of the first week via pathways that have been described as either apoptotic, autophagic, or necrotic [19, 20, 22, 23]. Granule cell death follows as a consequence of the loss of their primary neuron target population, the Purkinje cells: by the end of the second postnatal month, almost all Purkinje cells and 90% of the granule cells have degenerated in the +/*Lc* cerebellum [24]. We have previously hypothesized that before cell death pathways are activated in +/*Lc* Purkinje cells, the chronic depolarization caused by the constitutive cation leak current from the GluR*δ*2^*Lc*^ channel stresses the neuron's ion exchange and energy production systems [25, 26]. In support of this hypothesis, we have previously shown that cytochrome oxidase activity is dramatically increased in +/*Lc* Purkinje cells through P25 [26] and calcium levels are elevated in +/*Lc* Purkinje cells *in vitro* [27]. In addition, the distal dendrites of +/*Lc* Purkinje cells contain numerous dilated mitochondria, suggesting mitochondrial dysfunction [28]. +/*Lc* Purkinje cells also contain significantly higher levels of nitric oxide synthase (NOS) activity and protein nitration [25]. Stimulation of mitochondrial activity in response to the chronic depolarization is likely to increase the production of reactive oxygen species (ROS) and increases in intracellular Ca^++^ will stimulate NO production. NO and *O*_2_^−^ free radicals will react to form peroxynitrite, a highly reactive oxidizing agent which can damage and kill cells by tyrosine nitration of critical proteins and membrane lipid peroxidation [29, 30]. The results of this study demonstrate that the expression of the Na/K pump catalytic *α*3 subunit is increased, or at least more concentrated, in individual +/*Lc* Purkinje cells through P25. However, as +/*Lc* Purkinje cells degenerate by P25, total expression levels of *α*3 and its 40 kD breakdown product in the +/*Lc* cerebellum declines compared with WT cerebella. The density of active Na/K pumps assayed *ex vivo* is not altered at P15, near the peak of +/*Lc* Purkinje cell death, and decreased at P25 when there are relatively few remaining +/*Lc* Purkinje cells. Our interpretation of the results is that while the expression of the *α*3 subunit may be increased within individual +/*Lc* Purkinje cells in response to the chronic stress, potential increases in overall ATPase pump activity and ATP consumption due to increased expression of the *α*3 subunit may be limited by the deactivation and/or degradation of many *α*3 subunits.

## 2. Experimental Procedures

### 2.1. Animals

GluR*δ*2^+/*Lc*^ mutant and GluR*δ*2^+/+^ wild type (WT) pups were generated by mating B6CBACa A^w-J^/A-Grid2^*Lc*^/J males with WT females (C57BL/6J), both from Jackson Laboratories. All animals were housed in standard conditions (14 hours light, 10 hours dark) in the animal facilities at the Maryland Psychiatric Research Center and provided with food and water *ad libitum*. Males were harem mated with one male per two females. The day of birth was counted as postnatal day 0 (P0). The animal facilities are fully accredited by the American Association for the Accreditation of Laboratory Animal Care (AAALAC) and the studies were conducted in accordance with the Guide for Care and Use of Laboratory Animals provided by the NIH.

Mice were either euthanized by cardiac perfusion with 0.9% saline followed by 4% paraformaldehyde (while deeply anesthetized with Euthasol, >100 *μ*g/g) or by decapitation. Following the perfusions with 4% paraformaldehyde, brains were removed from the skull, postfixed for 2 hours, and then cryoprotected with 20% sucrose in 10 mM phosphate buffed saline (PBS). At least 48 hours later, the fixed brains were embedded in OCT and frozen in isopentane. Following decapitation, freshly dissected brains were either bisected and frozen in aluminum foil for Na/K pump activity assays or the cerebellum was isolated and frozen in crushed dry ice for Western blot analysis.

### 2.2. Immunohistochemistry

Fixed, frozen brains were cut at 12 *μ*m on a Leica cryostat, collected directly on slides, and stored at −70°C until stained. For immunofluorescence studies, slides were rinsed in 10 mM PBS, followed by incubation in two changes of 0.1 M glycine for 5 min each. Endogenous fluorescence was reduced by incubating the sections in 50 mM ammonium chloride for 1 hour. The sections were then rinsed three times in 10 mM PBS and then incubated for an hour in blocking solution containing 3% normal goat serum and 0.3%Triton X-100. Sections were then incubated in the primary antibodies overnight at 4°C. Sections were double labeled with either rabbit polyclonal anti-*α*1 (gift of Dr. M. Blaustein) or rabbit polyclonal anti-*α*3 Na/K ATPase (Upstate, now Millipore, 1/500: and mouse monoclonal anti-calbindin (Sigma, 1/5000) or rabbit polyclonal anti-activated caspase-3 (R&D, 1/500) and monoclonal mouse anti-*α*3 Na^+^/K^+^ ATPase (ABR Affinity BioReagents, Thermo Scientific Pierce Antibodies, 1/250). The sections were rinsed 3 times in PBS and then incubated for 2 hours with fluorescent-labeled secondary antibodies (anti-mouse or anti-rabbit Alexa 594 and Alexa 488, Molecular Probes, 1/200). After incubation, they were rinsed once in 10 mM PBS and incubated with 300 nM DAPI, then rinsed 3 times in 10 mM PBS, once in distilled water, and coverslipped with gel mount. The finished slides were then photographed using either an Olympus FV500 laser scanning confocal microscope or a Zeiss Axioplan fluorescence microscope. Confocal digital images were cropped and adjusted using Adobe Photoshop for color balance and intensity. All immunofluorescence experiments included slides (WT and +/*Lc*) with no 1antibody incubation as a control for nonspecific immunolabeling. The specificity of both the mouse monoclonal (ABR Affinity BioReagents) and the rabbit polyclonal (Upstate/Millipore) anti-*α*3 antibodies was also tested with Western blots using homogenized cerebellar tissue from WT and +/*Lc* mutant mice (data not shown). The ABR mouse monoclonal antibody labeled a single 110 kD band that represents the unbound, native *α*3 isoform, while the Upstate rabbit polyclonal antibody labeled two bands, the 110 kD *α*3 isoform and a 40 kD band that has been shown to be a byproduct of *α*3 isoform cleavage by calpain activity [31].

For the semiquantitative comparison of Na/K ATPase immunolabeling in WT and +/*Lc* cerebella, digital images of *α*3 isoform and calbindin immunolabeling of Purkinje cells were taken at 40x on a Zeiss Axioplan with an Olympus DP70 CCD camera within the first two days after staining all of the sections (to avoid uneven fading artifacts). The raw, unprocessed images were then analyzed using Metamorph Version 7.0r1. Threshold and colocalization functions were used to selectively measure the intensity of *α*3 isoform immunolabeling in areas that colocalized with calbindin-stained Purkinje cells in the same section. At least 3 molecular layer regions per cerebellum were selected from vermal sections in each WT or +/*Lc* cerebella and the mean value calculated for each cerebellum. The number of WT and +/*Lc* cerebella analyzed at each time point was P10, *n* = 5 WT, 4 +/*Lc*; P15, *n* = 5 WT, 5 +/*Lc*; P25, *n* = 4 WT, 5 +/*Lc*. The intensity of the *α*3 isoform fluorescence signal at P10, P15, and P25 is expressed as a percent change from labeling intensity in control Purkinje cells at P10.

#### 2.2.1. Western Blot Analysis

WT and +/*Lc* cerebella were collected from freshly dissected brains, rapidly frozen in dry ice and stored at −70°C until processed. Each cerebellum was homogenized in a buffer containing 50 mM Tris (pH 8.0), 150 mM NaCl, 5 mM EDTA, 1%SDS, 10 *μ*L/mL Protease inhibitor cocktail (Sigma), 1 mM PMSF, and 1 mM NaVO_4_. The homogenate was centrifuged at 15,000 rpm for 15 minutes. Protein concentration in the supernatant was measured using a BioRad protein assay kit. Protein extracts were diluted in Laemmli sample buffer with *β*-mercaptoethanol and denatured at 37°C for 5 min and 10 *μ*g of protein per well was resolved on a Tris-glycine gel. Protein was transferred overnight at 4°C onto a PVDF membrane. The membrane was rinsed with 5% nonfat dry milk dissolved in 1 × TBS. It was then incubated in the anti-*α*3 Na/K ATPase (ABR, 1/1000 or Upstate, 1/5000), diluted in TBS/0.1% Tween (TBS-T) with 1% milk overnight at 4°C, and then rinsed 3 × 10 min each with PBS-T. The sample membrane was incubated in alkaline phosphatase-conjugated secondary antibody (diluted 1/1000) and the protein was detected using Bio-Rad immun-Star chemiluminescence kit. Film exposed to the chemiluminescent signal was digitized using a light box and Pixera Pro150ES digital camera connected to a Power Macintosh. The optical density of the images was calibrated using a photographic calibration step tablet (Kodak) so that data is collected in the linear range of the O.D. The relative density of the labeled protein bands was determined using densitometric measurements with ImageJ. In all studies, once data from the antigen of interest had been collected, the membrane was stripped and labeled for total protein with India ink as a loading control. The density of *α*3 isoform protein bands was corrected for the total amount of protein by calculating the ratio of the density of the *α*3 isoform band and the total protein. Changes in the density of the corrected *α*3 isoform band were expressed as a percent of the averaged density of the *α*3 isoform bands from P25 controls within the same gel.

### 2.3. *In Situ* Na/K ATPase Activity Assay

*Histochemistry*. The density of active Na/K pumps was measured in frozen tissue sections using a procedure modified from previously published protocols [32, 33]. WT and +/*Lc* brains were bisected and rapidly frozen in dry ice for 2 min. The brains were then cut the same day in parasagittal sections at 20 *μ*m. A kidney from one of the wild type animals was also frozen and a 20 *μ*m section of kidney was placed on each slide as a positive control. Two slides with 2 sections per slide from each brain were used in the assay. The sections were fixed for 10 min in 2% paraformaldehyde, rinsed 3 times each in PBS, rinsed twice in 50 mM Tris/100 mM sucrose (pH 7.4), and then rinsed 3 times for 15 min each time in Tris/sucrose buffer. They were then incubated for 20 min at 37°C in a lead citrate mix pH 8.8 of 4mM Potassium Citrate, 4 mM Lead Nitrate, 250 mM Glycine, 25 mM potassium hydroxide, 20% DMSO, 10 mM para-Nitrophenylphosphate (p-NPP), and 2.5 mM Levamisole. Sections were then rinsed in distilled water, rinsed for 10 min in TRIS/sucrose buffer, and rinsed again 2 times in distilled water. Sections were developed for 2 min in 1% ammonium sulfide, rinsed 2 times in distilled water, rinsed for 10 min in PBS on a rocker, rinsed in distilled water, covered in crystal mount, and allowed to dry at room temp. As an assay for ouabain insensitive activity, one slide from each brain was treated with 10 mM Ouabain during the final Tris/sucrose incubation and the lead citrate incubation.

*Quantification of Enzyme Activity*. Stained slides were photographed at 5x on a Leica DMR microscope with a 24-megapixel Power Phase Scanning camera (Phase 1, Inc.). To decrease variations in background illumination, images of both the sections and adjacent blank areas of slide were photographed. The background images were subtracted from the section images using MatLab. Because of the large number of sections to analyze in each histochemical experiment, digital images were collected over three consecutive days. To calibrate for any potential differences between images taken in different sessions (e.g., changes in light levels), the change in average density of a selected section that was imaged all three days was subtracted from the respective day. Using ImageJ, a systematic random set of points was selected to measure optical densities. The density for the molecular layer and granular cell layers was averaged for each brain from measurements of two sections per brain per experiment. The ouabain-specific activity was calculated by subtracting the density of the ouabain treated tissue from the density of the untreated tissue.

## 3. Statistical Analyses

Statistical comparisons between experimental and control groups were made using two-way or one-way analysis of variance (ANOVA) and post hoc comparisons were made using Bonferroni/Dunn (Statview 5.01).

## 4. Results

### 4.1. Cellular Distribution of *α*3 Na/K Pump Isoforms in WT and +/*Lc* Purkinje Cells

**Figure 1:**
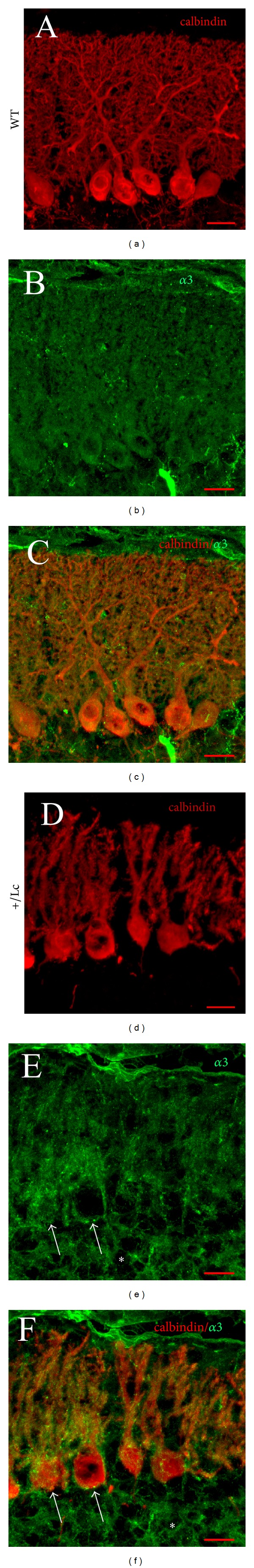
Confocal images of immunohistochemistry for the *α*3 isoform of Na/K pumps in the cerebellar cortex of wild type (A)–(C) and +/*Lc* mice (D)–(F) at P15. The cerebellar sections in (A)–(F) were double labeled for calbindin (red) and the *α*3 isoform (green) with overlaid images in (C) and (F). The white arrows in (E) and (F) indicate calbindin labeled +/*Lc* Purkinje cells with particularly intense *α*3 isoform immunostaining. The asterisks in (E) and (F) indicate *α*3 isoform labeling in the granule cell layer. Scale bars: 20 *μ*m.

A previous study has shown that in an adult mouse cerebellum, the *α*1 Na/K pump subunit is expressed in granule cells and glomeruli, *α*2 subunits are expressed in astrocytes, and *α*3 subunits are expressed in Purkinje cells, basket cell processes, and mossy fiber glomeruli [7]. Since both Purkinje and granule cells degenerate in the +/*Lc* mutant, the *α*1 and *α*3 subunits were initially selected for study as the most likely isoforms to show altered expression patterns. A preliminary immunohistochemical survey did not show any evidence of changes in the distribution or intensity of immunolabeling of the *α*1 subunit (results not shown), but there was evidence of distinct changes in the pattern of immunolabeling for the *α*3 subunit in the +/*Lc* cerebellum from P10 to P25. Vermal cerebellar sections from at least 4 WT and +/*Lc* cerebella at P10, P15, and P25 were double immunolabeled for calbindin and the *α*3 subunit using the Upstate rabbit polyclonal antibody that recognizes both the 110 kD full length *α*3 isoform and the 40 kD byproduct of *α*3 isoform cleavage in Western blots. In both WT and +/*Lc* cerebella from P10 to P25, the *α*3 subunits appear to be expressed in Purkinje cells, the molecular layer, and granule cell layer glomeruli in a similar pattern to that described by Peng et al. [7] in the cerebellum of adult rats. *α*3 expression in WT Purkinje cells is diffusely distributed throughout the dendrites and cell body as shown for P15 WT Purkinje cells in Figures 1(b) and 1(c) (green labeling). In contrast, the immunolabeling for *α*3 is qualitatively different at all three ages in +/*Lc* Purkinje cells. From P10, the *α*3 isoform labeling becomes more intense and less diffuse within +/*Lc* Purkinje cells. As shown for P15 +/*Lc* Purkinje cells in Figures 1(e) and 1(f), immunolabeling for the *α*3 subunit (green) is more punctate and concentrated around the primary dendrites and cell bodies so the labeling appears more intense, though there is some variation between different Purkinje cells. For example, the *α*3 labeling is particularly intense in the two left most +/*Lc* Purkinje cells in Figures 1(d)–1(f) (white arrows), where the *α*3 isoform appears to surround the stunted primary Purkinje cell dendrites especially in the lower two-thirds of the molecular layer. *α*3 immunolabeling is also seen around the Purkinje cell bodies but not as much within the cell body in comparison with WT Purkinje cells. *α*3 immunolabeling in the granule cell layer glomeruli is indicated by white asterisks in Figures 1(e) and 1(f). While we cannot rule out the possibility that some of the increased *α*3 immunolabeling in the Purkinje cell and molecular layers in the +/*Lc* cerebellar cortex may be in climbing fibers, it is clear that the intensity and distribution of *α*3 immunolabeling is altered in the +/*Lc* cerebellar cortex.

**Figure 2:**
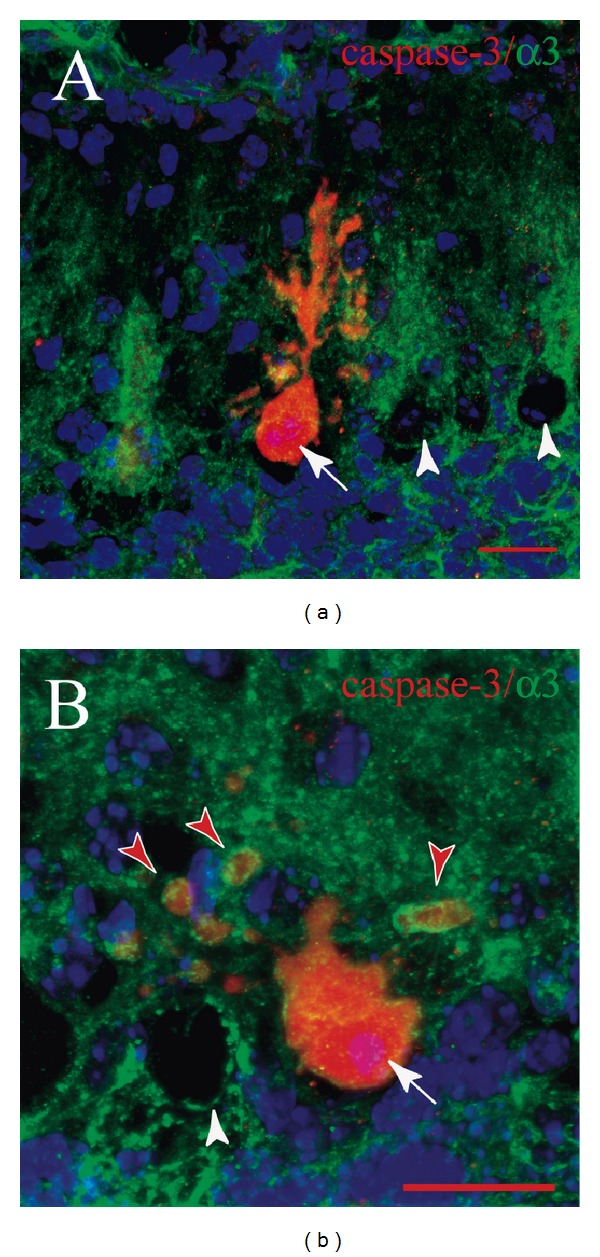
Confocal images of immunohistochemistry for activated caspase-3 (red), the *α*3 isoform (green), and DAPI (blue) in +/*Lc* cerebellar sections at P15. The white arrows in (a) and (b) indicate degenerating +/*Lc* Purkinje cells that are also immunolabeled for the *α*3 isoform. The red arrowheads indicate what appear to be the degenerating dendrites of the +/*Lc* Purkinje cell surrounded by *α*3 immunolabeling. The white arrowheads indicate the cell bodies of +/*Lc* Purkinje cells that have not yet started to express activated caspase-3. The nuclear DAPI labeling is faint and diffuse in the neighboring +/*Lc* Purkinje cells that do not yet express activated caspase-3. The monoclonal mouse anti-*α*3 isoform specific only for the full 110 kD *α*3 isoform was used in this case and a similar labeling pattern is observed compared to the rabbit polyclonal *α*3 antibody. Scale bars: 20 *μ*m.

+/*Lc* Purkinje cells that appear to be in the final stages of degeneration express activated caspase-3 [34, 35]. To determine if *α*3 subunit expression persists in dying Purkinje cells, cerebellar sections from P15 and P25 +/*Lc* cerebella were double labeled with antibodies to activated caspase-3 and the *α*3 isoform and counterstained with DAPI. The distribution of *α*3 isoform labeling in activated caspase-3 positive +/*Lc* Purkinje cells was examined in cerebellar sections from three +/*Lc* mutants at P15. Confocal images of two such degenerating +/*Lc* Purkinje cells are shown in Figures 2(a) and 2(b), illustrating that +/*Lc* Purkinje cells continue to express the *α*3 isoform even as they degenerate. The stunted dendrites and cell body of the activated caspase-3 positive (red) +/*Lc* Purkinje cell shown in Figure 2(a) show punctate labeling for *α*3 (green and yellow) throughout the remaining dendritic tree and cell body. The +/*Lc* Purkinje cell shown in Figure 2(b) appears to be at an advanced stage of degeneration with only a few retracted dendrites remaining as detached spherical blebs or tubes (red arrowheads). Yet the remaining parts of the degenerating Purkinje cell dendrites are surrounded by *α*3 immunolabeling. DAPI labeling (purple) in the nuclei of both activated caspase-3 positive neurons shows the condensation of the nuclei in the degenerating +/*Lc* Purkinje cells (white arrows). Nuclear DAPI labeling is found in the neighboring +/*Lc* Purkinje cells that have not yet begun to express activated caspase-3 (white arrowheads).

**Figure 3:**
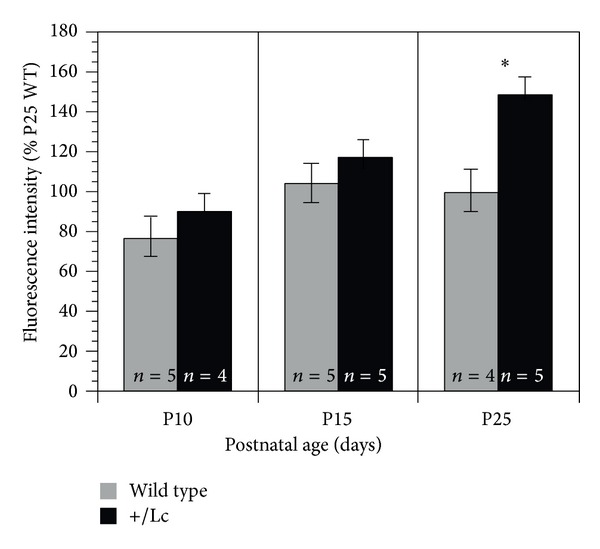
Densitometric measurements of the intensity of immunofluorescent labeling for the *α*3 subunit in the WT and +/*Lc* cerebellar cortex. The graph illustrates the relative changes in fluorescence intensity relative to the intensity of Purkinje cell immunolabeling in the P25 WT cerebellum (P10, *n* = 5 WT, 4 +/*Lc*; P15, *n* = 5 WT, 5 +/*Lc*; P25, *n* = 4 WT, 5 +/*Lc*).

The qualitative descriptions of the developmental changes in immunolabeling for the **α*3* isoform suggest that it is diffusely distributed throughout the Purkinje cell dendritic tree from P10 to P25 in the WT cerebellum. In contrast, in +/*Lc* Purkinje cells, *α*3 immunolabeling becomes more intense from P10 to P25 with punctate labeling concentrating along the Purkinje cell primary dendrites and cell bodies. As an assay for developmental changes in *α*3 immunolabeling, the intensity of the *α*3 (green) fluorescence signal coincident with calbindin labeling in WT and +/*Lc* Purkinje cells was measured using MetaMorph (Figure 3). Two-way ANOVA analysis of the fluorescence intensity measurements indicates that there are significant age- and genotype-dependant effects in *α*3 subunit immunofluorescence between WT and +/*Lc* Purkinje cells (Figure 3: Age: ANOVA *F*_2,22_ = 7.3, *P* = 0.0038; genotype: ANOVA *F*_1,22_ = 7.3, *P* = 0.0128), but the age *x* genotype interaction does not reach significance (ANOVA *F*_2,22_ = 1.85, *P* = 0.18). Post hoc analyses of the complete data set suggest that there are significant increases in *α*3 fluorescence intensity between P10 and P15 and between P10 and P25 and an overall significant difference between fluorescence intensity in +/*Lc* and WT Purkinje cells (Bonferroni/Dunn; *P* < 0.0167). A further exploratory post hoc one-way ANOVA analysis separating the data set by age suggests that while differences between *α*3 isoform immunofluorescence in +/*Lc* and WT Purkinje cells at P10 and P15 may contribute to the main effect, they are not significantly different when analyzed separately (P10 one-way ANOVA *F*_1,7_ = 2.22, *P* = 0.18; P15 one-way ANOVA *F*_1,8_ = 0.48, *P* = 0.5). However, there is a significant difference between the intensity of *α*3 isoform immunolabeling in +/*Lc* and WT Purkinje cells at P25 with higher levels in the +/*Lc* Purkinje cells (ANOVA *F*_1,7_ = 7.45, *P* < 0.03).

### 4.2. Analysis of *α*3 Isoform Protein Expression Levels

**Figure 4:**
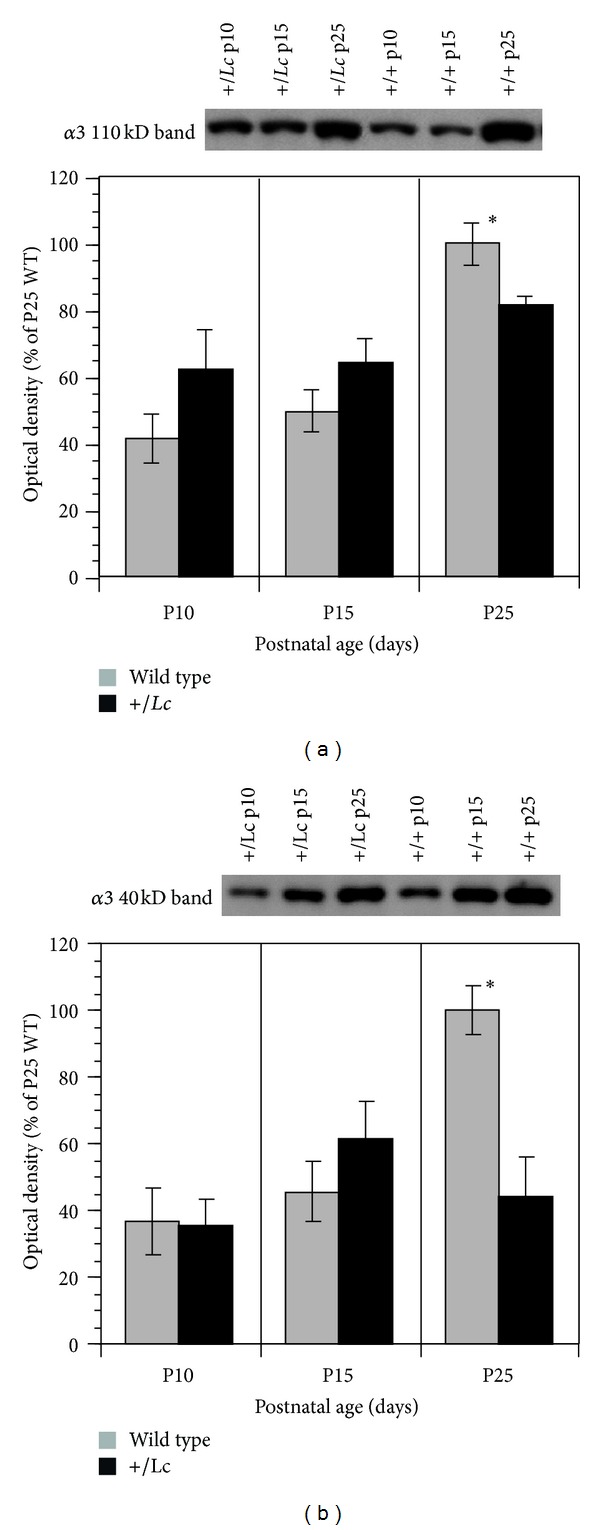
Expression levels of the *α*3 subunit 110 kD and 40 kD protein bands at P10, P15, and P25 in WT and +/*Lc* cerebella as determined by densitometry of Western blots. Representative Western blots are shown for the *α*3 110 kD (a) and 40 kD bands (b) at P10, P15, and P25, along with graphs of the relative density of the bands expressed as a percentage of the mean band intensity for P25 WT cerebella.

The increase in *α*3 immunofluorescence in +/*Lc* Purkinje cells from P10–P25 may be due to a variety of reasons, including either a redistribution of existing **α*3* isoforms or an increase in the expression levels of the *α*3 isoform. To distinguish between these possibilities, protein extracts of cerebellar tissue were prepared from WT and +/*Lc* cerebella at P10, P15, and P25 and the relative levels of *α*3 isoform expression were quantified by Western blot (Figure 4). Western blots were performed with both the mouse monoclonal (ABR Affinity BioReagents) and the rabbit polyclonal (Upstate/Millipore) anti-*α*3 antibodies. The ABR mouse monoclonal antibody labeled a single 110 kD band that represents the unbound, native *α*3 isoform, while the Upstate rabbit polyclonal antibody labeled two bands, the 110 kD *α*3 isoform and a 40 kD band that has been shown to be a byproduct of *α*3 isoform cleavage by calpain activity [31]. The Upstate rabbit polyclonal 110 kD band showed more background than the ABR mouse monoclonal, so the mouse monoclonal antibody was used to measure *α*3 isoform levels and the rabbit polyclonal antibody was used to measure levels of the 40 kD *α*3 isoform calpain degradation product. Representative images of Western blots for the 110 kD *α*3 isoform are shown for +/*Lc* and WT cerebella at P10, P15, and P25 in Figure 4(a).  Figure 4(b) shows representative *α*3 40 kD degradation product bands for WT and +/*Lc* cerebella at P10, P15, and P25.

Quantitative changes in the relative optical densities of the 110 kD *α*3 isoform protein and 40 kD degradation product are shown in the graphs in Figures 4(a) and 4(b), respectively. Two-way ANOVA of the 110 kD protein density data indicates that there is a significant effect of age (ANOVA *F*_2,37_ = 10.241, *P* = 0.003), but there are no significant effects of genotype (ANOVA *F*_1,37_ = 1.32, *P* = 0.26) or age *x* genotype interactions (ANOVA *F*_2,37_ = 1.83, *P* = 0.18). Post hoc exploratory analyses indicate that in the +/*Lc* and WT cerebella, *α*3 isoform protein levels at P25 are significantly higher than at P10 and P15 (Bonferroni/Dunn, *P* < 0.0167). A separate one-way ANOVA analysis of the data separated by age indicates that *α*3 isoform protein levels are only significantly different between WT and +/*Lc* cerebella at P25 (ANOVA *F*_1,12_ = 5.18, *P* = 0.042). The results indicate that *α*3 isoform levels in the WT and +/*Lc* cerebellum increase with age after P15, but by P25 the increase in *α*3 levels in +/*Lc* cerebella is not as great as in WT cerebella.

Two-way ANOVA of the 40 kD *α*3 isoform protein levels (Figure 4(b)) indicates that there are significant effects of age (ANOVA *F*_2.43_ = 6.33, *P* = 0.004), genotype (ANOVA *F*_1,43_ = 8.61, *P* = 0.0053), and age *x* genotype interactions (ANOVA *F*_2,42_ = 3.8, *P* < 0.028). Post hoc analyses indicate that group levels for the *α*3 40 kD fragment are significantly higher at P25 compared with P10 levels (Bonferroni/Dunn, *P* < 0.0167) but not between P25 and P15 or P15 and P10 (Bonferroni/Dunn, *P* > 0.0167). One-way ANOVA separated by age suggests that the genotype effect is primarily due to a significant difference between the amount of *α*3 40 kD fragment at P25 (ANOVA *F*_1,14_ = 15.33, *P* = 0.0016), with significantly lower levels in +/*Lc* cerebella. There are no significant differences between levels of the 40 kD fragment at P10 or P15. The results suggest that by P25 there is a dramatic increase in the 40 kD *α*3 fragment in WT cerebella, but this age-related increase is not matched in the +/*Lc* cerebella.

### 4.3. Histochemical Measurements of Active Na/K Pump Density

**Figure 5:**
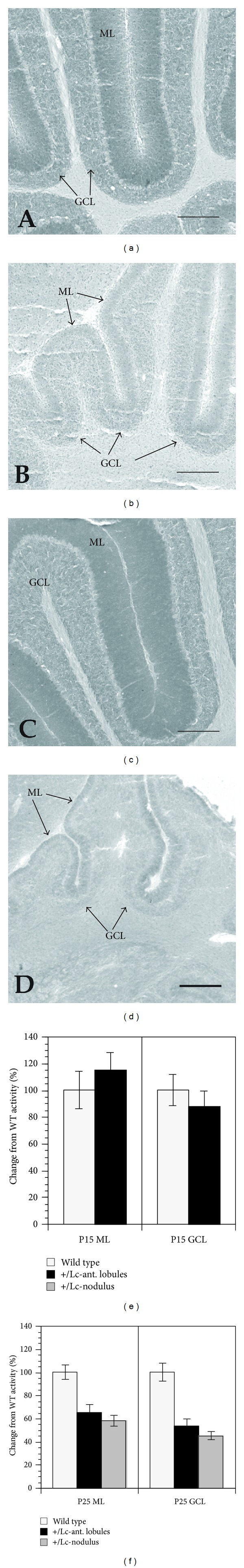
Histochemical assay for ouabain-sensitive Na/K pump activity in P15 (a), (b) and P25 (c), (d) WT and +/*Lc* cerebellar sections. The results of densitometric measurements of the molecular (ML) and granule cell layers (GCL) are expressed as a percent of wild type activity levels either at P15 (e) or P25 (f). At P25, densitometric measurements were made in both anterior lobules of the cerebellum and the nodulus. Scale bars: 200 *μ*m.

Since the GluR*δ*2^*Lc*^ channel mediates an Na^+^ leak current we hypothesized that the density of active Na^+^/K^+^ ATPase pumps would be increased from P10 through P25 in +/*Lc* Purkinje cells in response to increased intracellular Na^+^ levels. Furthermore, the changes in the distribution of the catalytic *α*3 subunit of the Na/K pump also suggest that the distribution and density of pump activity would be altered in +/*Lc* Purkinje cells. To assay changes in the distribution of active Na/K pumps in the +/*Lc* cerebellum, p-NPP was used as a substrate in an *in situ* histochemical assay to detect ouabain-sensitive, potassium-dependent activity of the Na/K pump complex. Representative images of histochemical labeling for Na/K pump activity in WT and +/*Lc* cerebellar cortex at P15 and P25 are shown in Figure 5. The images of both the wild type and +/*Lc* cerebella are taken at the same magnification, but the +/*Lc* cerebella is smaller at P15 and dramatically reduced in size by P25 compared to WT because of ongoing (and by P25, extensive) +/*Lc* Purkinje and granule cell death. Quantitative analysis of *in situ* ouabain-sensitive Na/K pump activity in histological sections of wild type and +/*Lc* cerebellar sections at P15 and P25 shows that the overall density of active Na/K pump in the molecular and granule cell layers is within WT levels at P15 (ML; ANOVA *F*_1,9_ = 0.467, *P* > 0.1; GCL; ANOVA *F*_1,9_ = 0.47, *P* > 0.1) but decreases by P25 in both the +/*Lc* molecular layer ( ML: ANOVA *F*_3,16_ = 11.9, *P* < 0.003) and granule cell layer (GCL: ANOVA *F*_3,16_ = 19.4, *P* < 0.001; Figures 5(e) and 5(f)). *In situ* active Na/K pump density is expressed as a percent of WT values at either P15 or P25. Cerebellar slices from WT and +/*Lc* mice at P15 and P25 were processed on separate days so it was not possible to compare densitometric density between P15 and P25.

At P15, significant numbers of +/*Lc* Purkinje cells are still present, so we assume that the Na/K pump activity measurements in the molecular layer reflects pump activity in Purkinje cells dendrites in addition to other cellular elements in the molecular layer. Therefore, at P15 all of the measurements were made in the molecular and granule cell layers of lobes III and IV as representative areas. However, by P25 most +/*Lc* Purkinje cells have degenerated in anterior and central lobules, but approximately 40 to 50% of the +/*Lc* Purkinje cells persist in the nodulus [36]. To determine if there is a difference in Na/K pump activity between cerebellar regions with few Purkinje cells versus regions with surviving Purkinje cells, Na/K pump activity measurements at P25 were made in the molecular and granule cell layers of lobules III and IV versus X (the nodulus) in control and +/*Lc* mutant cerebella (Figure 5(f)). Following the activity measurements, the same sections were stained with cresyl violet to verify the presence of +/*Lc* Purkinje cells. While only a few Purkinje cells were found throughout lobules III and IV, the density of surviving Purkinje cells in the regions of the nodulus analyzed for Na/K pump activity was noticeably higher (data not shown). However, the preferential survival of +/*Lc* Purkinje cells in the nodulus did not appear to affect the overall decline in Na/K pump activity observed at P25 in the +/*Lc* cerebella in both the molecular and granule cell layers.

## 5. Discussion

The Lurcher mutation in GluR*δ*2 converts this enigmatic glutamate receptor into a constitutively open cation channel that chronically depolarizes +/*Lc* Purkinje cells as they mature following the first week of postnatal development [20, 22]. The cation leak in +/*Lc* Purkinje cells is thought to initiate excitotoxic cell death pathways [19]. One assumption of excitotoxic mechanisms is that the ionic imbalance resulting from overstimulation of glutamate receptors and the resulting influx of Na^+^ and Ca^++^ ions places overwhelming stress on cellular energy metabolism systems [17, 18]. In +/*Lc* Purkinje cells, in particular, mitochondrial cytochrome oxidase activity is significantly increased, possibly in response to increased cellular energy requirements [26]. The goal of this study was to test the hypothesis that Na^+^/K^+^ ATPase *α*3 isoform expression and the density of active Na/K pumps are increased in +/*Lc* Purkinje cells. This increase would be consistent with an increased demand for ATP production (and increased cytochrome oxidase activity) to counterbalance the GluR*δ*2^*Lc*^ Na^+^ and Ca^++^ leak current. However, the results indicate that while the expression of the catalytic *α*3 isoform may be increased in individual +/*Lc* Purkinje cells, the density of active Na/K pumps is not significantly increased above wild type levels in the cerebella of younger +/*Lc* mutants (P10 to P15). Furthermore, by 25, overall expression levels of the *α*3 isoform and its 40 kD breakdown product are decreased in the +/*Lc* cerebellum compared to WT, along with a decrease in the density of active Na/K pumps. The lower cerebellar expression levels of the *α*3 isoform and decreased density of active Na/K pumps by P25 in the +/*Lc* cerebellum may simply reflect the substantial loss of +/*Lc* Purkinje and granule cells by this age. While the results indicate that the density of active Na/K pump units are not significantly increased in +/*Lc* Purkinje cells in response to the leak current, it is important to note that in this study we have not analyzed the *in vivo* unit activity of Na/K pumps in living +/*Lc* Purkinje cells.

### 5.1. Expression of the Na/K Pump *α*3 Subunit in Depolarized +/*Lc* Purkinje Cells

In this study, initial immunohistochemical studies of the expression pattern of the primary subunits of the Na/K pump expressed in cerebellar Purkinje cells, the *α*3, and *β*1 isoforms suggested that only the expression of the catalytic *α*3 subunit is altered in the +/*Lc* cerebellar cortex. The change in expression of the *α*3 isoform in +/*Lc* Purkinje cells was subsequently analyzed by fluorescent immunolabeling to provide information about subunit localization. The significant increase in the intensity of immunofluorescence for the *α*3 subunit from P10 through P25 (Figure 3) in +/*Lc* Purkinje cells indicates either that there is an increase in the density of the protein or that the localization of the subunit is altered within each +/*Lc* Purkinje cell as their dendrites and cell bodies degenerate. The Western blot data shows that the total cerebellar levels of both the native *α*3 subunit and its 40 kD cleavage product are not significantly altered from P10 to P15 in the +/*Lc* cerebella compared to WT and their levels do not subsequently increase through P25 as in WT cerebella. While both immunofluorescence and Western blot techniques provide important complementary qualitative and quantitative information about the pattern of protein expression, the results need to be interpreted with caution because of their inherent limitations. The significant increase in the intensity of immunofluorescence for the *α*3 subunit in +/*Lc* Purkinje cells suggests that there is an increase in the density of the protein within individual Purkinje cells. However, it is not possible to distinguish between the possibilities that there is an increase in the expression of the *α*3 subunit or that the localization of the subunit is altered as +/*Lc* Purkinje cells fail to differentiate (especially their dendrites) and eventually degenerate. The Western blot data appears to favor the latter hypothesis since the only significant difference in *α*3 expression is a relative decrease at P25 in +/*Lc* cerebella when 70–90% of the +/*Lc* Purkinje cells and 60% of the granule cells have degenerated [24, 36]. It is also important to consider that +/*Lc* Purkinje cells are degenerating from at least P10 onwards with approximately 50% of the +/*Lc* Purkinje cells missing by P13 to P15 [24]. Given that the intensity of immunolabeling for the subunit within each +/*Lc* Purkinje cell steadily increases from P10 through P25 (Figure 3), it is possible that *α*3 protein levels may actually steadily increase within individual +/*Lc* Purkinje cells, but the overall expression levels in the cerebellum may not significantly change because of the ongoing loss of +/*Lc* Purkinje cells. Thus, even at P25, *α*3 subunit expression levels may be significantly increased within the stunted dendritic trees characteristic of +/*Lc* Purkinje cells, but overall cerebellar *α*3 levels may have declined overall because most Purkinje cells have died by this time.

While the immunohistochemistry and Western blot data are consistent with a change in the density of Na/K pumps within individual Purkinje cells, these results do not indicate how these changes may translate into alterations in pump activity. A previous study of adult acutely dissociated rat thalamic neurons depolarized by exposure to veratridine or monensin found that the resulting Na^+^ influx resulted in an increase in Na/K pump density and in the level of phosphorylated pump molecules (implying increased pump activity; [37]). In the chronic Na^+^ leak in developing +/*Lc* Purkinje cells in this study, the evidence suggests that the density of active Na/K pumps is not altered in the +/*Lc* cerebellar cortex until after P15. At P15, there is no significant difference in the density of histochemically measured, ouabain-sensitive active Na/K pumps between the molecular layers of wild type and +/*Lc* cerebella. By P25, there is a significant decrease in the density of molecular layer active Na/K pumps, both in anterior lobules with virtually no Purkinje cells or the nodulus with relatively higher numbers of surviving +/*Lc* Purkinje cells.

The diffuse pattern of Na/K pump activity in the +/*Lc* cerebellar cortex is in stark contrast to the distribution of cytochrome oxidase (COX) histochemical staining at the same ages [26]. Individual +/*Lc* Purkinje cells at P15 and P25 stain darkly for COX, filling the entire Purkinje cell body and dendritic tree. The dramatic increase in COX activity suggests that the mitochondrial respiratory pathway is upregulated to produce more ATP. If the density of active Na/K pumps had been increased in response to the chronic Na^+^ influx in +/*Lc* Purkinje cells it seems reasonable to expect that the intensity of the histochemical labeling would have increased along Purkinje cell dendritic membranes. However, the staining pattern for Na/K pump activity remained diffuse in the +/*Lc* cerebellar cortex, with no evidence of discrete patches of labeling corresponding to the patchy labeling for *α*3 subunit observed in fluorescently labeled sections. The discrepancy between the distribution of *α*3 subunits detected by immunofluorescence and the pattern of Na/K pump histochemical activity could be due to limitations in the spatial resolution of the histochemical staining or some of the *α*3 immunolabeling labeling could represent deactivated Na/K pumps, including the 40 kD *α*3 cleaved isoform. In a mouse model of focal cerebral ischemia, no changes were found in protein or mRNA levels for Na/K pump isoforms [12], but the level of Na/K pump activity was decreased along with a change in ouabain sensitivity, indicating that the decrease in activity was due to intrinsic modifications of the ATPase pump.

There are a number of mechanisms that can reduce or irreversibly block Na/K pump activity. Na/K pump isoforms are routinely recycled by internalization in lysosomes and degradation by lysosomal cathepsin D [31, 38]. However, the membrane bound Na/K pump complex can also be inactivated by the calpain-mediated cleavage of the scaffolding protein, ankyrin, that binds the Na/K pump to the membrane skeleton [39] or by degradation of Na/K pump isoforms themselves [31]. When oxidized, Na/K pump isoforms become more sensitive to intracellular proteinases and activated calpain will cleave the *α*3 isoform into a 40 kD degradation product [31]. In this study, the amount of the 110 kD native *α*3 subunit and 40 kD degradation product increases in WT cerebella through P25 suggesting that a significant fraction of the native *α*3 subunit is oxidized and degraded by calpain in normal cerebellar tissue. Calpain has recently been shown to be active in individual, isolated +/*Lc* Purkinje cells at P14 based on immunolabeling for the 136 kD fragment of *α*-spectrin cleaved by calpain [19]. In this study, despite the loss of significant numbers of +/*Lc* Purkinje cells at P15, there is no significant reduction in the levels of the 40 kD band in +/*Lc* cerebella until P25, when most Purkinje and granule cells have degenerated. As in the case of the 110 kD native *α*3 subunit, it is possible that the amount of the 40 kD degradation is increased in individual +/*Lc* Purkinje cells at P15 and P25, but total amounts are within control levels at P15 and significantly decreased at P25 because of the ongoing +/*Lc* Purkinje cell loss. +/*Lc* Purkinje cell degeneration is associated with an increase in oxidative stress, including an increase in mitochondrial cytochrome oxidase activity, which may generate more reactive oxygen species as a byproduct of increased respiratory activity. The results do not indicate that there is a dramatic increase in oxidized forms of the *α*3 subunit by P15 in the +/*Lc* cerebellum since there is not a significant increase in levels of the 40 kD byproduct, but the results are consistent with sustained proteolysis of the *α*3 subunit by calpain in +/*Lc* Purkinje cells until most have degenerated.

Na/K pump activity is also regulated by nitric oxide (NO)-cGMP [10] or NO-PKG pathways [40] and inhibited by peroxynitrite through the formation of nitrotyrosine or the modification of cysteine residues [15, 16]. Previous studies of +/*Lc* Purkinje cells have shown that their degeneration is associated with increased expression of neuronal nitric oxide synthase and nitrotyrosine [25]. Since the Na/K pump is sensitive to peroxynitrite and NO, we hypothesize that while the cation leak increases the expression of the catalytic *α*3 isoform, interactions with peroxynitrite, NO/PKG, and/or NO/cGMP reduce the density of active Na/K pumps. A reduction in the density of active Na/K pumps in +/*Lc* Purkinje cells may contribute to a Na^+^ overload in these neurons and exacerbate the cell death process, since increases in intracellular Na^+^ have been associated with induction of apoptosis [5, 41].

## 6. Conclusion

The analysis of the distribution and activity of the Na/K pump in this study of degenerating +/*Lc* Purkinje cells demonstrates that while the expression and/or distribution of the *α*3 subunit is disrupted in the cerebellar cortex of the +/*Lc* mutant, there is no evidence to suggest that the density of active pumps is increased in response to the constitutive influx of Na^+^ ions through the mutant GluR*δ*2^*Lc*^ channels. We had hypothesized that Na/K pump activity would be stimulated as a homeostatic response to the Na^+^ ion influx. The lack of evidence for an increased density of functional Na/K pumps does not rule out the possibility that the activity of individual pumps is increased within each +/*Lc* Purkinje cell, thereby stimulating an increased demand for ATP which would eventually lead to depletion of cellular energy resources. The Na/K pump is estimated to use up to 50% of the ATP produced by neurons [42]. Na^+^ is normally the rate-limiting factor in Na/K pump activity [43–45] and elevations in the concentration of Na^+^ within +/*Lc* Purkinje cells may increase the activity of individual pumps because of a more complete saturation of its Na^+^ binding sites. Thus, while there may be an increased ATP consumption in +/*Lc* Purkinje cells because of the increased activity of individual pumps, this study suggests that any increase in Na/K pump activity and ATP consumption is not due to an overall increase in the density of active ATPase pumps in chronically depolarized Purkinje cells.
